# Targeting phosphoglycerate kinases by tatridin A, a natural sesquiterpenoid endowed with anti-cancer activity, using a proteomic platform

**DOI:** 10.3389/fmolb.2023.1212541

**Published:** 2023-09-11

**Authors:** Giusy Ferraro, Antonia Voli, Matteo Mozzicafreddo, Federica Pollastro, Alessandra Tosco, Maria Chiara Monti

**Affiliations:** ^1^ Department of Pharmacy, Università di Salerno, Fisciano, Italy; ^2^ PhD Program in Drug Discovery and Development, Department of Pharmacy, Università di Salerno, Fisciano, Italy; ^3^ Department of Clinical and Molecular Sciences, Università Politecnica Delle Marche, Ancona, Italy; ^4^ Department of Pharmaceutical Sciences, Università Del Piemonte Orientale, Novara, Italy; ^5^ PlantaChem Srls, Novara, Italy

**Keywords:** sesquiterpenes, functional proteomics, PGK1, CXCR4, gastric cancer, cancer dissemination

## Abstract

Tatridin A (TatA) is a germacrane sesquiterpenoid containing one E-double bond and one Z-double bond in its 10-membered ring, which is fused to a 3-methylene-dihydrofuran-2-one moiety. Tatridin A bioactivity has been poorly investigated despite its interesting chemical structure. Here, a functional proteomic platform was adapted to disclose its most reliable targets in leukemia monocytic cells, and phosphoglycerate kinases were recognized as the most affine enzymes. Through a combination of limited proteolysis and molecular docking, it has been discovered that tatridin A interacts with the active domains of phosphoglycerate kinase 1, altering its hinge region, and it can be accountable for tatridin A inhibition potency on enzyme activity. A more detailed tatridin A biological profile showed that it is also fully active against gastric cancer cells, downregulating the mRNA levels of chemokine receptor 4 and β-catenin and inhibiting the invasiveness of living KATO III cells as a direct consequence of phosphoglycerate kinase 1 antagonism.

## 1 Introduction

Sesquiterpene lactones are a widespread group of bioactive plant secondary metabolites: the scientific interest in this class of compounds has emerged due to their vast biological activity which is useful for human health. Chemically, they are characterized by a 15-carbon backbone containing an α,β-unsaturated carbonyl moiety and a conserved α-methylene-γ-lactone. Some of the numerous recognized activities for sesquiterpene lactones comprise anti-microbial, anti-fungal, anti-viral, anti-tumor, anti-malarial, anti-diabetic, analgesic, and anti-inflammatory properties (Matos et al., 2021).

**FIGURE 1:**
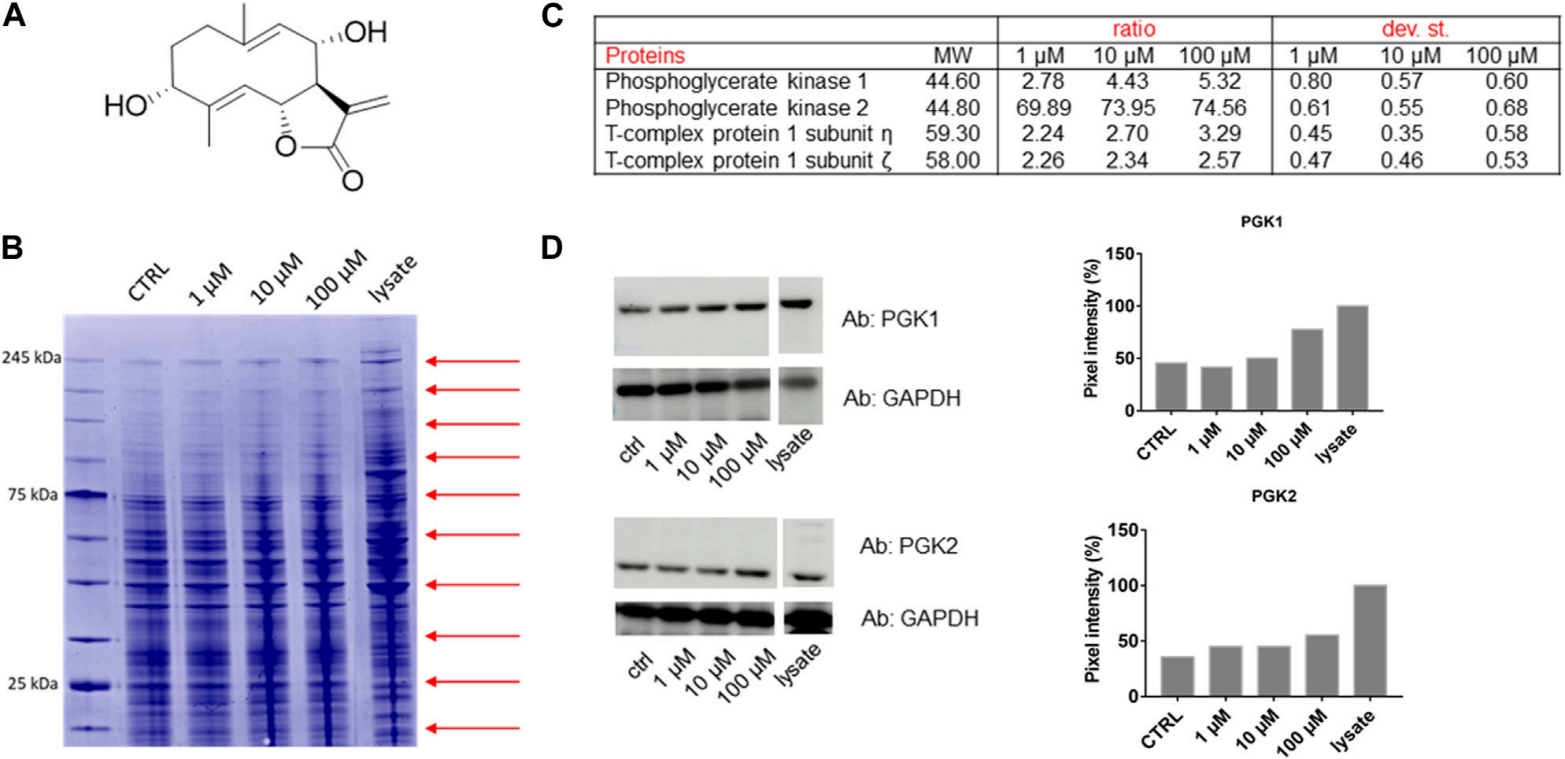
**(A)** Chemical structure of Tatridin A (TatA). **(B)** Coomassie-stained gel showing the protection of proteins to protease upon TatA interaction (red arrows indicate the cutting sites). **(C)** Proteome Discoverer-retrieved protection ratio of the four proteins shared among all DARTS experiments at three TatA concentrations. **(D)** Immunoblotting analysis of one DARTS experiment revealing PGK1 and PGK2 protected in a TatA concentration-dependent fashion, together with the densitometric analysis. GAPDH is resistant to subtilisin under these experimental conditions and is used as the loading control.

Among the sesquiterpene lactones, tatridin A (TatA, Figure 1A), isolated for the first time from the aerial parts of *Anthemis melanolepis* (Saroglou et al., 2010), showed an *in vitro* anti-microbial potential against Gram-positive bacteria, such as *Bacillus cereus*, *Micrococcus luteus*, and *Staphylococcus aureus*, and a certain *in vitro* cytotoxic activity against a panel of human tumor cell lines. In particular, TatA showed cytotoxicity against myeloid leukemia cell lines (Rivero et al., 2003).

To deepen the understanding of the TatA anti-cancer action mechanism, a combination of drug affinity responsive target stability (DARTS) and targeted limited proteolysis assisted by multiple reaction monitoring mass spectrometry (t-LIP-MRM) has been applied on a human leukemia monocytic cell line (THP-1), chosen as a model system. DARTS and t-LIP-MRM are based on the evidence that when a protein binds to a small molecule, it undergoes conformational changes and becomes a more stable structure less prone to unspecific controlled proteolysis. This resistance helps us in distinguishing, in a complex lysate, proteins interacting with a molecule from the proteins more abundant at their own molecular weight in the treated samples, as detectable by traditional bottom-up proteomics (Ceccacci et al., 2020; Morretta et al., 2020; Ren et al., 2021). Then, to monitor the altered proteolytic peptides, we used limited subtilisin proteolysis followed by extensive tryptic digestion, generating a mixture of semi-tryptic peptides (due to the subtilisin-processed regions) and fully tryptic peptides (due to the undigested peptides). The fully tryptic peptides can be easily quantified by MRM events and result in higher areas if limited proteolysis is less effective due to small molecule protection.

Both the described strategies were applied to TatA, and they indicated a strong interaction between TatA and phosphoglycerate kinases, especially the isoform 1 named phosphoglycerate kinase 1 (PGK1); thus, we deeply explored this using a multidisciplinary approach combining *in* *silico*, *in vitro*, and *in cell* experiments.

Indeed, we were interested in this protein–ligand interaction since PGK1 has different functions besides its metabolic function. For instance, PGK1 acts in regulating angiogenesis, and its overexpression promotes gastric cancer cell invasiveness (Liu et al., 2022a). Thus, a deep *in cell* investigation has been carried out using KATO III as a model of gastric cancer cells to prove the mechanism of action of the selected natural compound.

## 2 Materials and methods

### 2.1 TatA PAMPA assay

A donor solution (50 μM) was prepared by diluting 5 mM TatA stock solutions using phosphate-buffered saline (PBS, pH 7.4). The experiments were performed as reported in Colarusso et al. (2023). The permeability value Log Pe was determined.

### 2.2 Cell cultures

THP-1 cells (human monocytic cell line derived from an acute monocytic leukemia patient) and KATO III cells (gastric carcinoma derived from a metastatic site, poorly differentiated) were obtained from the American Type Culture Collection (ATCC, Manassas, VA, United States). They were preserved in RPMI 1640 (Euroclone, #ECM2001L, Italy), supplemented with 20% (v/v) fetal bovine serum (FBS, Euroclone, #ECS0180D, South America, origin EU approved) for KATO III cells and 10% for THP-1 cells and penicillin–streptomycin solution (100 U/mL penicillin and 100 μg/mL streptomycin) (Euroclone, #ECB3001D, Italy), and were grown at 37°C with 5% CO_2_ in a humidified system.

### 2.3 TatA cellular targets by DARTS

THP-1 cells were lysed in M-PER™ (Mammalian Protein Extraction Reagent, Thermo Scientific, Waltham, Massachusetts, United States) supplied with the protease inhibitor cocktail (Sigma-Aldrich, Darmstadt, Germany). The lysate was centrifuged at 10,000 g for 10 min at 4°C, and the Bradford assay was used to determine the protein concentration of the obtained supernatant. Then, DARTS experiments were conducted as follows: different amounts (1 μM, 10 μM, and 100 μM) of TatA were incubated with 300 µg of THP-1 cell lysate for 1 h at room temperature. Then, the samples were submitted to limited proteolysis for 30 min at 25°C, at a ratio of 1:1500 w/w of subtilisin (Sigma-Aldrich, Darmstadt, Germany) with respect to the protein amount. Two samples of cell lysates were treated with dimethyl sulfoxide (DMSO) and one of them with subtilisin as control experiments. Then, the protease was quenched by adding PMSF (phenylmethylsulfonyl fluoride, Sigma-Aldrich, Darmstadt, Germany, 1 mM final concentration) to each sample. Then, all the samples were boiled in Laemmli buffer (60 mM Tris-HCl, pH 6.8, 2% SDS, 0.001% bromophenol blue, 1% glycerol, and 2% β-mercaptoethanol), and 20 μg of the samples were loaded on a 4%–12% Bis-Tris Criterion™ XT Precast Gel (Bio-Rad Laboratories S.r.l., Hercules, California, United States), which was then stained with a Coomassie solution and submitted to a densitometric analysis through ImageJ. This experiment was repeated in triplicate. Protein bands were excised from the gels and submitted to an *in situ* tryptic digestion protocol. Briefly, gel slices were reduced with DTT (1,4-dithiothreitol), alkylated with IAA (iodoacetamide), and washed and rehydrated on ice for 1 h in 12 ng/μL trypsin solution. Then, the excess enzyme was removed and replaced with ammonium bicarbonate (AmBic; 50 mM; pH 8.5), allowing protein digestion to proceed overnight at 37°C. Subsequently, supernatants were collected, and peptides were extracted from each gel slice and shrunk in 100% ACN (acetonitrile). The peptide mixtures were dried under a vacuum and dissolved in formic acid (FA, 10%) for the LC-MS/MS analysis. Then, 1 µL of each sample was injected into a nano-UPLC (Ultra-High-Pressure Liquid Chromatography) system (Thermo Scientific, Waltham, Massachusetts, United States), separating peptides on an EASY-Spray PepMap^TM^ RSLC C18 column (3 µm; 100 Å; 75 μm × 50 cm; Thermo Scientific, Waltham, Massachusetts, United States) at a flow rate of 0.3 nL/min. MS data were acquired using a Q Exactive classic mass spectrometer (Thermo Scientific, Waltham, Massachusetts, United States), provided with a nano-electrospray (nanoESI) source. Subsequently, database searches were carried out on Proteome Discoverer, employing the SwissProt database and the following parameters: maximum of two missed cleavages, trypsin digestion, and carbamidomethyl (C) as the fixed modification; oxidization (M) and protein N-terminal acetylation as variable modifications; and MSPepSearch was used to perform a spectral library search with a mass tolerance of 10 ppm for MS1 and 0.02 Da for MS2.

### 2.4 Validation of DARTS results via immunoblotting

The samples were then submitted for Western blotting analysis (Morretta et al., 2021a) with monoclonal antibodies against PGK1 and phosphoglycerate kinase 2 PGK2 (1:1000) and against glyceraldehyde 3-phosphate dehydrogenase (GAPDH, 1:2500).

### 2.5 PGK1 MRM method building

PGK1 (UniProt accession: P00558) tryptic peptides were selected through the proteomic data resource PeptideAtlas (https://db.systemsbiology.net/sbeams/cgi/PeptideAtlas) on its human build and queried into the complete human SRMAtlas build (https://db.systemsbiology.net/sbeams/cgi/PeptideAtlas/GetTransitions) to recover their most intense fragments. Thus, full MRM methods reporting PGK1 peptides and their best transitions were gained and searched in a THP-1 lysate tryptic digest. The experiment was conducted as reported in Morretta et al. (2021b).

Thus, a global MRM method comprising 16 transitions was obtained, allowing 60% PGK1 mapping.

### 2.6 t-LIP-MRM analysis

THP-1 cell lysates were raised with or without TatA at 1 μM and 10 µM, respectively, for 1 h at 25°C. The samples were then submitted to limited proteolysis with a 1:500 (w/w) ratio of subtilisin, and the experiment was conducted as reported in Morretta et al. (2021b). To guarantee the exact injection volume of different samples in the LC-MS system and, thus, accurate quantification of tryptic peptides in different runs, the [Val5]-angiotensin II peptide was spiked in all samples and monitored using the following MRM transition: Q1 at m/z of 516.0 and Q3 at m/z of 263.0 corresponding to the doubly charged peptide and its y_2_ fragment.

### 2.7 Molecular docking analysis

To structurally analyze the binding between TatA and PGK1, we performed molecular docking using the three-dimensional structures of the binding partners obtained from the PubChem database (Kim et al., 2021) with CID number 14466152 and the Protein Data Bank (Berman et al., 2000) with pdb ID 2wzb (Cliff et al., 2010) and processed as reported previously (Del Gaudio et al., 2018). Moreover, the prediction of binding between TatA and PGK2 was also performed using the very high/confident model of the enzyme (ID: AF-P07205-F1) obtained from the AlphaFold Protein Structure Database (Jumper et al., 2021). The molecular docking procedure was carried out on the SwissDock web server (Grosdidier et al., 2011), with a docking zone including the entire protein. All other parameters (docking type and flexibility) were set to default values. Additionally, this analysis was repeated simultaneously using AutoDock Vina, a genetic algorithm-based software application, with the same parameters. The energetically best complex was analyzed using the Protein-Ligand Interaction Profiler (PLIP) web service (Adasme et al., 2021) and rendered using PyMOL software (PyMOL Molecular Graphics System, version 2.0, Schrödinger, LLC., Cambridge, MA, United States). The stability of this complex was checked by performing a molecular dynamics (MD) analysis using GROMACS 2023.1 as previously reported in Mozzicafreddo et al. (2023) and following the simulation for 10 ns.

### 2.8 PGK1 activity assay

The experiment was performed both with human recombinant PGK1 (#268–11354, RayBiotech, Peachtree Corners, United States) and using THP-1 and KATO III cell lysates as a source of PGK1. More in-depth, THP-1 or KATO III cell lines were lysed in M-PER™ (Mammalian Protein Extraction Reagent, Thermo Scientific, Waltham, Massachusetts, United States) supplied with a protease inhibitor cocktail (final concentration 1×). The obtained proteome (centrifuged at 14,000 g; 4°C; 15’ in the Eppendorf Centrifuge 5424 R) was quantified by the Bradford spectrophotometric assay.

PGK1 at a final concentration of 4 nM or THP-1 and KATO III cell lysates (1 µg) were incubated with and without TatA (10 nM, 1000 nM, 5000 nM, 10000 nM, and 50000 nM), and the samples were diluted in PGK assay buffer (47 µL) and added to a reaction mix (50 µL for each well), containing PGK developer, ATP, NADH (50 mM), and PGK substrate. In the first step of this enzymatic assay, PGK1 converts 3-PG and ATP to 1,3-bisphosphoglycerate (1,3-BPG) and ADP, respectively. The nascent intermediate is detected via a series of enzymatic reactions, and the last reaction is the oxidation of NADH to NAD^+^, which can be easily measured (OD = 340 nm). All the samples were shaken for 30 min in a 96-multi-well plate (final volume of 100 µL/well). The experiment was performed in duplicate. The absorbance of the produced NAD^+^ was monitored in the kinetic mode for 60′ using the Multiskan GO spectrophotometer by Thermo Scientific (37°C, orbital shaking: medium intensity, and 10″ on/10″ off; λ = 340 nm; 1 scan/5’). The same experiments were performed using TatB with identical settings.

### 2.9 Cell viability assay

KATO III cells were grown in 96-well plates at a cell density of 1 × 10^4^ cells/well. After 24 h, the cells were raised for 72 h with TatA (from 3.125 to 400 µM). MTT assay was performed as reported by Bellone et al. (2021).

### 2.10 Quantitative real-time PCR

KATO III cells were seeded in 6-well plates at a cell density of 5 × 10^5^ cells/well. After 24 h, the cells were incubated for another 24 h in the presence of 100 µM TatA. Total RNA was extracted using TRIzol reagent (Invitrogen, #15596018, New Zealand) following the manufacturer’s instructions, and 1 µg of total RNA was retro-transcribed by M-MLV Reverse Transcriptase (GeneSpin S.r.l, #STS-MRT, Italy). The real-time PCR was performed using the QuantStudio™ 5 instrument (Thermo Scientific, Waltham, Massachusetts, USA). Appropriate concentrations of cDNA were employed for each gene in a 12 μL volume using Luna Universal qPCR Master Mix (New England Biolabs, #M3003, United States). The primer sequences used are described in Supplementary Table S1. Data from technical duplicates of three independent biological experiments were examined using the ΔΔCT method and HPRT1 as a reference gene.

### 2.11 Transwell invasion assay

KATO III invasiveness was investigated using the Transwell cell culture (12 mm diameter and 8.0 μm pore size; Corning Incorporated, United States). The upper chamber membranes were covered with Collagen, Type I solution from Rat Tail (Sigma-Aldrich, Darmstadt, Germany) and located in wells comprising the 10% FBS-supplemented medium. Cells were seeded at 1 × 10^5^/insert into the upper chambers in the serum-free medium. Treatment with 100 µM TatA was finalized in the upper chamber. After 24 h of incubation at 37°C in a 5% CO_2_–95% air-humidified atmosphere, filters were fixed with 4% p-formaldehyde for 10 min and then with 100% methanol for 20 min. Cells on the lower surface of the filter were stained with 0.5% crystal violet solution. The cells that migrated to the lower surface were counted in 12 random fields using the EVOS light microscope (10×) (Life Technologies Corporation).

### 2.12 Analysis of a putative TatA–PGK1 covalent complex by LC-MS analysis

PGK1 (5 µM in PBS at pH 7.5) was incubated for different time periods, 3 h and 16 h at 37°C, with 50-fold molar excesses of TatA. Each sample was analyzed on a Q Exactive classic (Thermo Fisher Scientific, Bremen) equipped with an UltiMate 3000 Ultra-High-Pressure Liquid Chromatography system and an ESI source. Briefly, the mixture was loaded on a C4 BEH300 ACQUITY column (1.7 µm; 100 × 2.1 mm; Phenomenex) and eluted by a linear gradient (15%–65%) of aqueous acetonitrile containing FA (0.1%) over a period of 25 min. Mass spectra were collected (m/z 600–3000).

## 3 Results

### 3.1 Tatridin A permeation by PAMPA assays

TatA behavior in the PAMPA assay has been studied to understand its permeability (expressed as −log Pe) through an artificial lipid bilayer (Le Roux et al., 2020). Under our experimental conditions, TatA presented an optimal tendency to traverse the bilayer *in vitro* with a −log Pe of 4.87 ± 0.01.

### 3.2 Identification of tatridin A cellular targets through DARTS

Frequently, the binding of a ligand to its receptor stabilizes the latter through a more stable conformational structure. This determines that the ligand–receptor complex is less susceptible to enzymatic proteolysis. Thus, subtilisin has been chosen as a low-specificity enzyme, and the entire THP-1 cell lysate has been submitted to its action both in the presence or absence of TatA. The resistance to the hydrolysis can be monitored by SDS-PAGE since the putative target intensity will be enhanced by TatA treatment, due to its induced stabilization, depending on the concentration. A classic bottom-up proteomic approach allows for protein identification.

Under our conditions, THP-1 cells were manually lysed using non-denaturing agents, and they were treated with increasing TatA concentrations and then exposed to subtilisin for the limited proteolysis events. The opportune controls were considered. Then, Coomassie blue staining for the visualization of proteins separated by SDS-PAGE and bottom-up proteomics were achieved (Figure 1B). Nano-UPLC-MS/MS runs were carried out followed by the search in Proteome Discoverer to determine protein identity (Figure 1C).

Among the putative-identified targets, PGK1 and PGK2 were designated as the most valid TatA receptors because they were well protected from enzyme action in all DARTS repeats. The interaction between TatA and its targets was then unambiguously re-soluted, loading all DARTS samples on immunoblotting using anti-PGK1 and PGK2 antibodies (Figure 1D). Indeed, by comparing the immunoblotting signals corresponding to undigested PGK1 and PGK2 (∼47 kDa bands), it is clear that the intact protein signal increases its intensity in accordance with the TatA concentration. An accurate densitometric analysis was carried out on the full-length PGK1 and PGK2 signals, using GAPDH as a loading normalizer. As reported in Supplementary Figure S1, immunoblotting analysis on the T-complex sub.η and sub.ζ did not reveal a TatA concentration-dependent protection on both putative receptors and thus not considered the reliable targets.

Phosphoglycerate kinase 1 is a high-concentration enzyme existing in the majority of cells playing a crucial role in glycolysis; its role is to bring ATP through the phospho-transference reversible reaction from 1,3-bisphosphoglycerate to MgADP to yield 3-phosphoglycerate (3-PG) and MgATP or *vice versa* (He et al., 2019). Differently, phosphoglycerate kinase 2 provides the energy essential for sperm motility, and it is encoded by an autosomal retrogene with limited expression in germ cells (Sawyer et al., 2008). Structural studies suggest that the PGK2 active site is located in the N-terminal domain, and it is essentially identical to that of the cytoplasmic PGK1 (Sawyer et al., 2008), whereas there are changes in the site located in the C-terminal domain, also deputed to interact with other partners.

In this paper, we focused on the PGK1 enzyme due to its higher ubiquitous expression in all cells and its chief role in cellular metabolism.

The PGK1 three-dimensional structure has been well understood; it is a monomeric protein comprising two domains, corresponding to N- and C-terminal regions, which are connected by a central helix hinge positioned from aa 187 to aa 201 (Bernstein et al., 1997). The N-terminal domain of PGK1 is the central part of the active site, and it recognizes 1,3-BPG or 3-PG, while the C-domain receives the nucleotide substrate ADP or ATP. Once the two co-substrates are located inside the active site, the central helix moves the enzyme into a closed productive conformation. Indeed, the enzyme shifts from its open form with an affinity for the substrates to its closed form, which is competent to transfer the phosphoryl group (Liu et al., 2022a).

### 3.3 Analysis of the interaction features of PGK1 binding to tatridin A by the t-LIP approach and by *in silico* analysis

To explore the TatA interaction profile with the PGK1 target, our t-LIP -MRM plan was applied (Feng et al., 2014). T-LIP-MRM allows us to identify the target/ligand crossing peptide(s) in a complex cell lysate, observing the structural variations due to TatA interaction. THP-1 native proteins were mixed with TatA, and then two proteases were sequentially added to the samples: the first was subtilisin which was incubated under native conditions, and then, the sample was denatured and trypsin was added. This consecutive sample handling produces a mixture of semi-tryptic and fully tryptic peptides, which can be easily quantified by targeted MRM-MS allowing protein primary sequence coverage (Supplementary Figure S2). The area below the signals of the fully tryptic peptides is indicative of the receptor structural variations induced by the ligand; it will be higher if subtilisin proteolysis is less operative due to the protection of protein conformation by TatA.

A preliminary *in silico* quest using bioinformatics tools, PeptideAtlas and SRMAtlas, was performed to establish the PGK1 MRM signals relative to fully tryptic peptides which were more informative to create the protein fingerprint. As a second step, THP-1 proteins were denatured and digested by trypsin to identify the occurring peptides together with their fragmentation by LC-MRM-MS. Next, THP-1 proteins were mixed with TatA (1 μM and 10 μM) and digested with subtilisin under precise conditions of time, temperature, and enzyme-to-protein (1:500 w/w) ratio. The addition of subtilisin was then ceased, urea was added to the samples for denaturation, trypsin was supplemented as necessary, and LC-MRM-MS runs were performed to measure the area of each PGK1 tryptic peptide.

**FIGURE 2:**
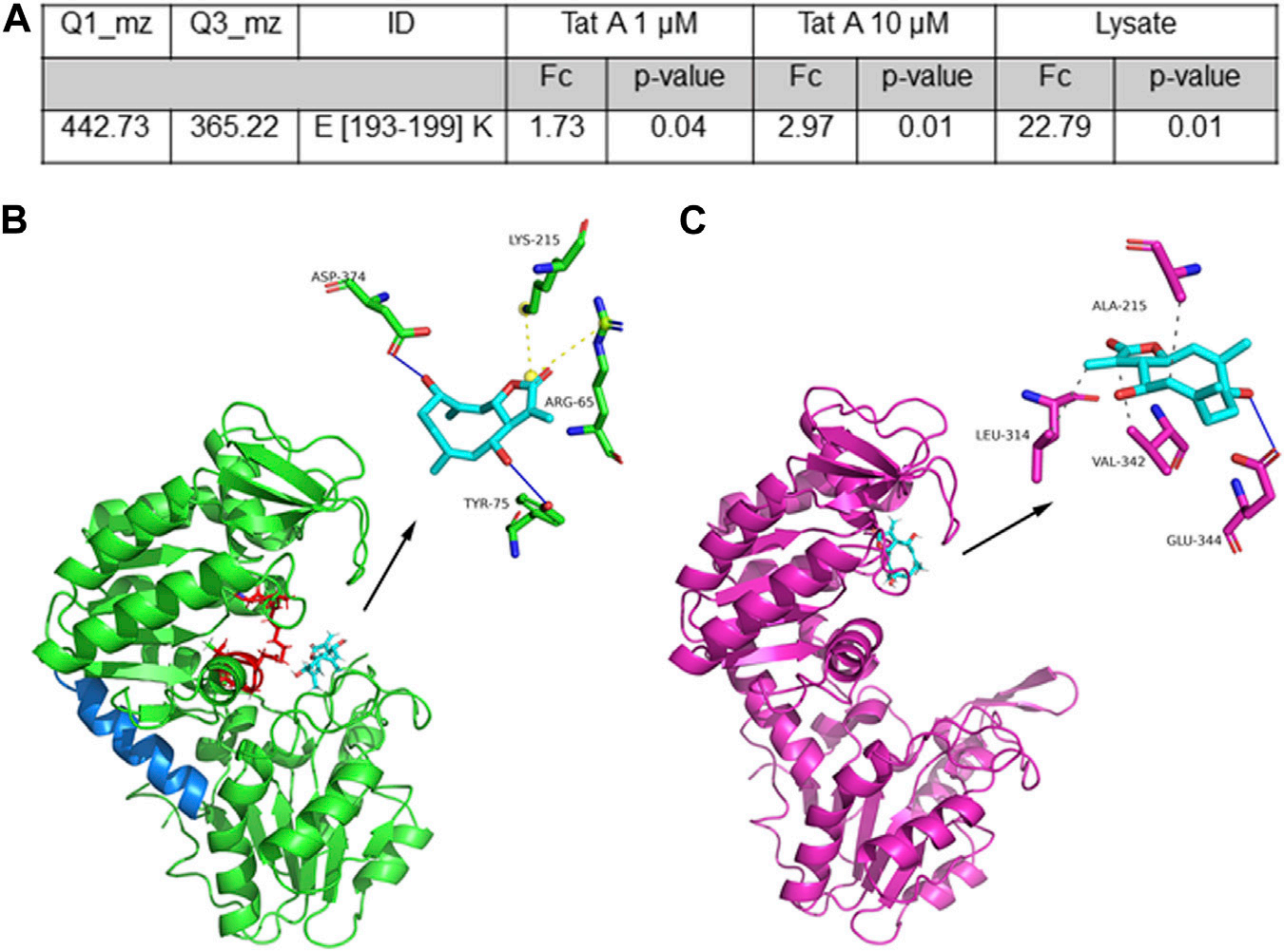
**(A)** Selected TatA peptide reported with its parent and daughter m/z value used in the MRM approach, its length, and the calculated fold change (FC and the associated *p*-value) as the ratio of the peptide area due to TatA protection. **(B)** Best predicted docking pose of TatA on PGK1. TatA is reported in light blue sticks, the PGK1 site for 3PG and ATP is reported in red (in particular, amino acids K215, G372, G373, D374, T375, G395, and G396), the hinge helix is reported in blue, and the amino acids involved in the interaction are reported in the 3D representation. **(C)** TatA is bound to PGK2.

Then, the data were analyzed by comparing the controls and the treated samples to disclose the direct or long-term conformational changes induced by TatA (Supplementary Figure S3). As reported in Figure 2A, there is a unique case in which, upon TatA incubation, the ratio of the tryptic peptide area is higher than 1.5 with a good significativity (*p*-value <0.05), and this peptide can be considered protected from proteolysis by the TatA action. Interestingly, only peptides 193–199 showed an increased intensity in the samples exposed to TatA in a concentration-dependent fashion, and it can be considered symptomatic of TatA protection. This peptide covers a large part of the central helix (aa from 187 to 201, Figure 2B in blue), which is fundamental for PGK1 activity, allowing the two active domains of the enzyme to approach each other during the catalytic cycle (Gondeau et al., 2008).

Molecular docking analysis of the TatA/PGK1 complex revealed a strong interaction, expressed with an equilibrium dissociation constant (*K*_*D,pred*_) of 5.52 μM. This value is comparable to the affinity of terazosin (TZN), an alpha-adrenergic blocker drug, for PGK1 (*K*_*D*_ = 2.78 μM), and to that obtained for the TatA/PGK2 complex (*K*_*D,pred*_ = 2.35 μM). These values are also comparable with those reported for DC–PGKI (*K*_*D*_ = 0.1 μM) (Bernstein et al., 1997) and ilicicolin H (*K*_*D*_ = 60 μM) (Bernstein et al., 1997), potent PGK1 antagonists. Parallel docking analysis was performed using AutoDock Vina to cross-check the results, and they were strongly in agreement with those reported previously; indeed, K_*D,pred*_ for the TatA/PGK1 complex was approximately 20 ± 9 µM and a root mean square distance (RMSD) between the geometries of the ligand, obtained with the two algorithms, was approximately 0.5 Å. The molecular dynamics analysis revealed that good stability (RMSD <1.5 Å) of the TatA/PGK1 complex is reached in 2.5 ns, a relatively fast time frame (Supplementary Figure S4). Structurally, as revealed in Figure 2B, TatA is able to form non-covalent interactions with Tyr75 and Asp374 (H-bonds) and with Arg65 and Lys215 (salt bridges) of PGK1. In particular, since Arg65 and Lys215 are located in the 3-phosphoglyceric acid (3PG) recognition site and Asp374 participates in the binding of ATP (Liu et al., 2022b), it can be stated that TatA is able to hinder the PGK1 active site accessibility to both substrates. Indeed, as widely reported in the literature, the 3-PG or 1,3-BPG binding site is present in the N-domain and the nucleotide-binding site is present in the C-domain (Liu et al., 2022b); thus, the substrates enter, and PGK1 shifts from open to closed conformation, causing a hinge bending movement that brings the groups together; and the transfer of the phosphoryl group occurs. In contrast, TatA/PGK2 and TZN/PGK1 binding sites are situated solely at the ATP binding site (Figure 2C). In this site, TatA could establish three hydrophobic interactions (with Ala215, Leu314, and Val342) and an H-bond (with Glu344). As reported in Supplementary Figure S5, PGK1 and PGK2 share high sequence identity even though many differences in terms of amino acid composition are encountered in the region between residues 310 and 340 and on residue 242, which is of key importance for ADP binding.

The results obtained from both LIP-MRM and molecular docking analysis propose that TatA directly interacts with PGK1 in a key region near 3PG and ATP binding sites as disclosed by molecular docking and that this binding is hardly able to alter the exposition of the hinge region as disclosed by LIP-MRM; both results point toward an alteration of enzyme activity due to TatA binding.

Moreover, PGK1 was incubated with 50× molar excess of TatA for both 3 h and 16 h, and LC-MS analyses were carried out to evaluate a possible covalent binding between the counterparts. As shown in Supplementary Figure S6, any increment in the PGK1 molecular weight was measured after incubation, and thus, no covalent adducts were disclosed by our analysis.

### 3.4 Tatridin A negatively affected PGK1 activity

**FIGURE 3:**
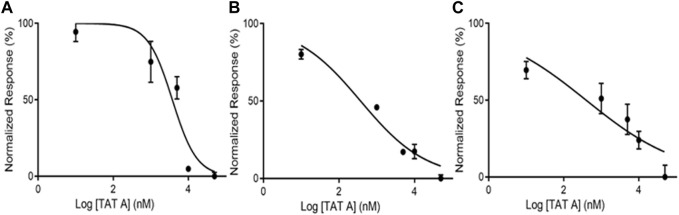
TatA is able to inhibit PGK1 activity on **(A)** the recombinant enzyme, **(B)** THP-1 cell lysate, and **(C)** KATO III cell lysate. The graphs were prepared using Prism software.

An *in vitro* assay was performed to discover the effect of TatA on PGK1 through the measurement of its activity in the presence of different concentrations of TatA, as reported by the manufacturer. Initially, the human recombinant PGK1 activity was monitored by carrying out the spectrophotometric assay in the kinetic mode in the presence of TatA at different amounts. As shown in Figure 3A, TatA modulates the activity of PGK1 in the negative mode, showing an IC_50_ value of 3760 ± 870 nM. Then, the same experiment was carried out using THP-1 and KATO III native cellular lysates as a source of PGKs in order to investigate whether, even under pseudo-physiological conditions, TatA was able to modulate PGK bioactivity. As expected and as reported in Figures 3B, C, TatA also inhibits the enzymatic activity in these more complex systems with a good potency profile and IC_50_ of 375 ± 112 nM and 397 ± 250 nM, respectively. The opportune control experiments were carried out as reported by the enzymatic kit manufacturer’s procedure, and, in addition, both the assays with PGK1 and cell lysates were performed in the presence of 1-epi-tatridin B, a germacranolide TatA analog. This molecule, which is inactive in many biological tests (Rivero et al., 2003), does not alter PGK1 activity at any tested concentration (Supplementary Figure S7).

### 3.5 TatA cellular activity on gastric carcinoma

To confirm the inhibitory activity of TatA in a cellular system, the gastric carcinoma cell line KATO III was also used. The cytotoxic activity of the natural compound was measured using an MTT assay on THP-1 as well as KATO III cells, and EC_50_ values of 38 ± 2 μM and 18 ± 4 µM were obtained after 72 h of incubation, respectively (Supplementary Figure S8).

**FIGURE 4:**
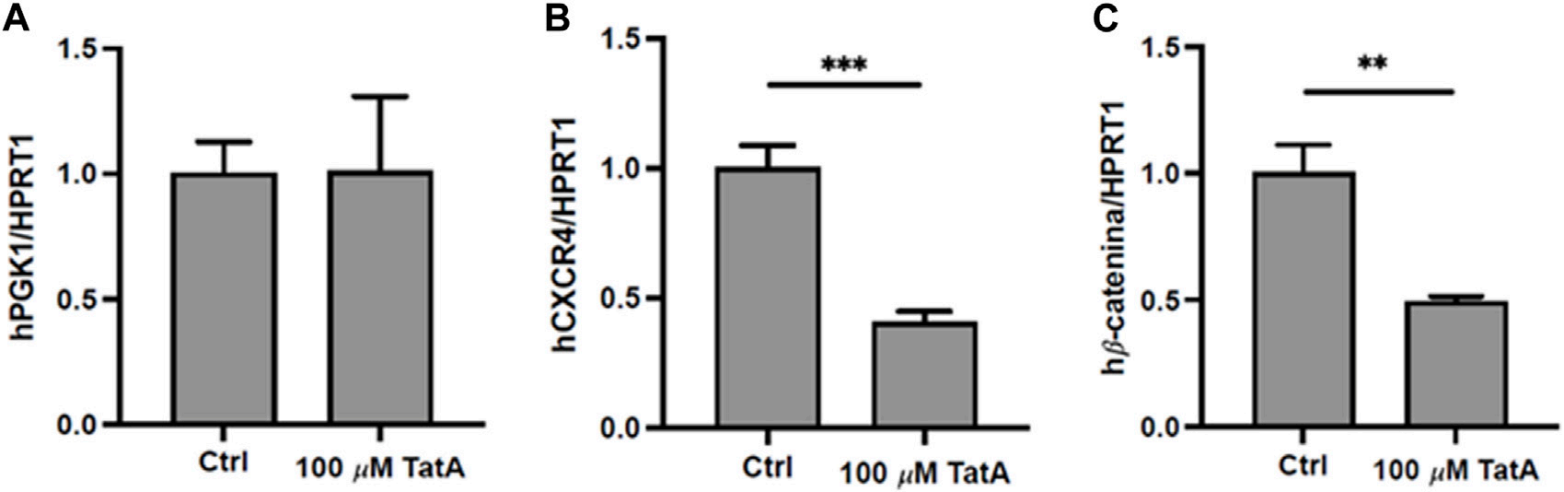
Quantitative real-time PCR (qRT-PCR) analysis of PGK1 **(A)**, CXCR4 **(B)**, and β-catenin **(C)** in KATO III cells after 24 h treatment with 100 μM TatA. HPRT1 was used as the housekeeping gene. Experiments were carried out in triplicate. Data are expressed as mean ± s.d. (*t*-test; **p-value ≤0.01, ***p-value ≤0.001).

Since PGK1 is described to influence CXCR4 and β-catenin expression in gastric cancer cells (Ziecker et al., 2010), promoting peritoneal carcinomatosis, KATO III cells were incubated with 100 µM TatA for 24 h, and CXCR4 and β-catenin expression was evaluated by RT-qPCR. Figure 4 shows that natural product administration determines a significant downregulation of both mRNAs, without altering PGK1 expression.

**FIGURE 5:**
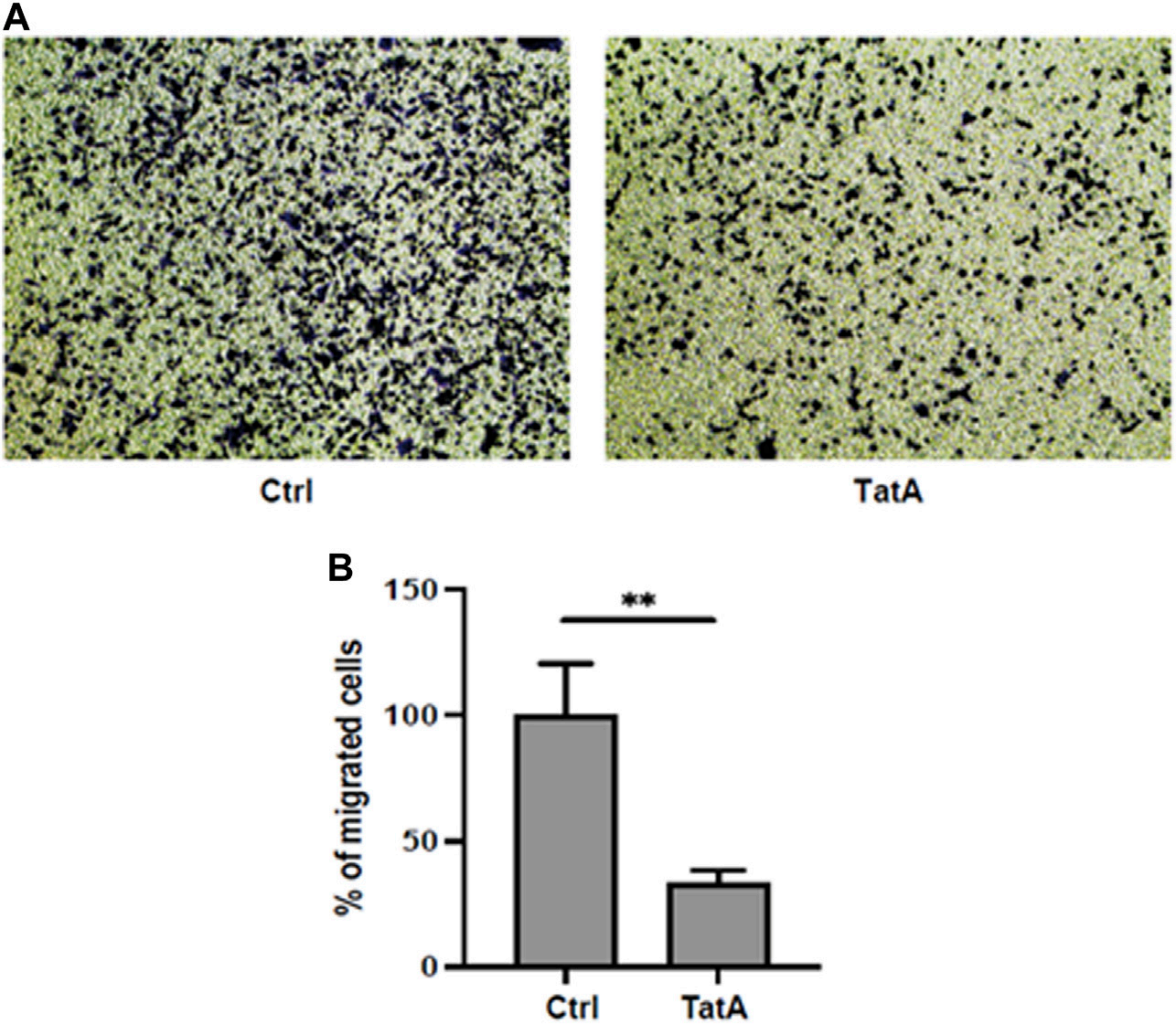
**(A)** Representative microscopic images of the bottom surface of Transwell filters stained with crystal violet (magnification ×10) showing KATO III cells treated with or without 100 μM TatA for 24 h. **(B)** Quantification of invasive cells. The data are shown as the mean number of cells per eight visual fields (magnification ×10) of three replicate wells ± s.d. (*t*-test; **p-value ≤0.01).

Moreover, PGK1 is also described to induce invasiveness in gastric cancer cells (Ziecker et al., 2010), and to explore whether our molecule could influence this activity, KATO III cells were incubated with 100 µM TatA for 24 h and a Transwell invasion assay was performed. As shown in Figure 5 A and B, TatA strongly reduces KATO III invasiveness, suggesting that PGK1 inhibition is also effective in a cellular context.

## 4 Discussion and conclusion

Phosphoglycerate kinases are essential enzymes that catalyze ATP production in aerobic glycolysis. In particular, isoform 1, also called PGK1, achieves several functions acting both as a metabolic enzyme and as a protein kinase that is competent to perform on different protein substrates (Zhang et al., 2018; Liang et al., 2020).

In addition to the regulation of metabolism, PGK1 takes part in numerous biological pathways, such as angiogenesis, autophagy, nucleic acid replication and repair, and development of tumor cells and their invasion, and is also linked with chemotherapy resistance and diagnosis of patients.

In different types of cancer, the *PGK1* gene acts as an oncogene to twitch cancer development, and it also acts as a key player in tumor metabolism parameter alteration through oncogenic signaling pathways (e.g., Akt/mTOR, Myc, Notch, and CXCR4/β-catenin). It has been stressed that, in different types of cancer, the speed of the conversion of glucose in pyruvate is 200 times higher than that in healthy cells and that, as a consequence, this phenomenon can endorse tumor invasion and migration (Akram, 2013; Zhang, 2020).

Many studies have demonstrated that PGK1 is upregulated in many types of human cancers, including kidney, endometrial, lung, and gastric cancer (Akram, 2013; Zhang et al., 2018; Zhang, 2020). The regulation of PGK1 activity includes ion regulation (Qian et al., 2019), nucleotide regulation (Zhang et al., 2020), redox state regulation (Zhou et al., 2019), and non-coding RNA regulation (Rojas-Pirela et al., 2020), whereas few synthetic or natural small molecules have been found to have an inhibitory effect on PGK1. For instance, a small molecule recently known to negatively affect its activity is NG52; it is reported that NG52 reduced the epithelial–mesenchymal transition and reversed the Warburg effect by inhibiting PGK1 activity in ovarian cancer or glioma both *in cell* and *in vivo* (Tsukamoto et al., 2013; Barros-Álvarez et al., 2014). Furthermore, dorsomorphin, MK-571, and LTP-10 were also identified in a wide screening as apparent inhibitors of PGK1 with IC_50_ values of 6.72, 25.24, and 2.30 μmol/L, respectively (Tsukamoto et al., 2013; Barros-Álvarez et al., 2014). They seem to be non-competitive inhibitors, different from TatA.

Moreover, aryl and alkyl bisphosphonates were found with the IC_50_ value of PGK1 activity inhibition at 0.84–200 μM (Kotsikorou et al., 2006), as well as some quinoxalines with the IC_50_ value at 1 nM, solely tested *in vitro* (Xu et al., 2012). In 2018, a cyclic sulfone compound, with an IC_50_ value at 17.15 μM was tested in cells (Bollong et al., 2018), and more recently, GQQ-792, a novel kind of epipolythiodiketopiperazine (EPT), obtained from the mangrove endophytic fungus *Tilachlidium sp*., has been found to be a covalent inhibitor of PGK1 *in vitro* and *in vivo* (Wang et al., 2021).

Here, our well-consolidated functional proteomic platform has been applied to a promising germacrane sesquiterpenoid, tatridin A, in order to investigate its role in cancer cells since the involvement of this molecule has been reported in the development of leukemia (Rivero et al., 2003). Our data pointed to a good interaction between TatA and PGK1 and a potent inhibition exerted by this small molecule on the enzyme activity.

Thus, prompted by our results, we next investigated TatA involvement in gastric cancers from a biological point of view since it was reported that PGK1 upregulation and/or its amplified expression rises the invasiveness of gastric cancer *in vitro* (Ziecker et al., 2010) and that targeting PGK1 in gastric cancer cells can modulate the chemokine receptor CXCR4 concentration and β-catenin, both involved in cancer progression.

Certainly, chemokines and their receptors play a relevant role in leukocyte movement and stimulation and cancer-related processes, such as angiogenesis and tumor growth. In particular, CXCR4 promotes the progression and scattering of several malignant tumoral pathologies, including prostate, non-small-cell lung, pancreatic, breast, and gastric cancer. Furthermore, β-catenin, a 92-kDa protein, is strongly related to cadherin-based cell adherence, as a downstream player in the Wnt-signaling pathway, and it is also a possible downstream receptor of PGK1. In gastric cancer, upregulation of the β-catenin expression well correlates with augmented proliferation, invasiveness, metastasis, angiogenesis, and drug resistance.

Our idea was to deeply understand one of the first natural compounds endowed with anti-cancer activity, targeting PGK1 in KATO III gastric cancer cells and showing a good potency in this cell line, and charmingly, it was found to be a non-covalent inhibitor.

As shown in the Results section, TatA works very well on KATO III cells, downregulating mRNA levels of the chemokine receptor 4 and β-catenin and inhibiting the invasiveness of living KATO III cells as a direct consequence of PGK1 antagonism.

TatA can be considered a novel and promising natural antagonist of PGK1 endowed with anti-cancer activity on gastric tumor cells, opening new avenues to deeply explore the class of bioactive sesquiterpenoid germacranolides.

## Data Availability

The proteomics data have been deposited in the ProteomeXchange Consortium via the PRIDE partner repository with the dataset identifier PXD034940. 6) Molecular docking structures were uploaded on Zenodo with DOI: 10.5281/zenodo.8124267.
