# Supplementary material for: Targeting phosphoglycerate kinases by tatridin A, a natural sesquiterpenoid endowed with anti-cancer activity, using a proteomic platform
Source: Front Mol Biosci. 2023 Sep 11;10:1212541. doi: 10.3389/fmolb.2023.1212541 (PMC10519794; doi:10.3389/fmolb.2023.1212541)
Supplement: Supplementary file 1 [file Table1.docx]

***Supplementary Material***

**Targeting Phosphoglycerate Kinases by Tatridin A, a natural sesquiterpenoid endowed with anti-cancer activity, by a proteomic platform.**

**Giusy Ferraro,^1,2^ Antonia Voli,^1,2^ Matteo Mozzicafreddo,^3^ Federica Pollastro,^4,5^ Alessandra Tosco^1^ and Maria Chiara Monti^1^***

^1^Department of Pharmacy, Università di Salerno, Fisciano, Italy

^2^PhD Program in Drug Discovery and Development, Department of Pharmacy, Università di Salerno, Fisciano, Italy

^3^Department of Clinical and Molecular Sciences, Università Politecnica delle Marche, 60126 Ancona, Italy

^4^Department of Pharmaceutical Sciences, Università del Piemonte Orientale, Novara, Italy,

^5^PlantaChem Srls, Novara, Italy

*****Corresponding author: Prof. Maria Chiara Monti email: [mcmonti@unisa.it](mailto:mcmonti@unisa.it)

**
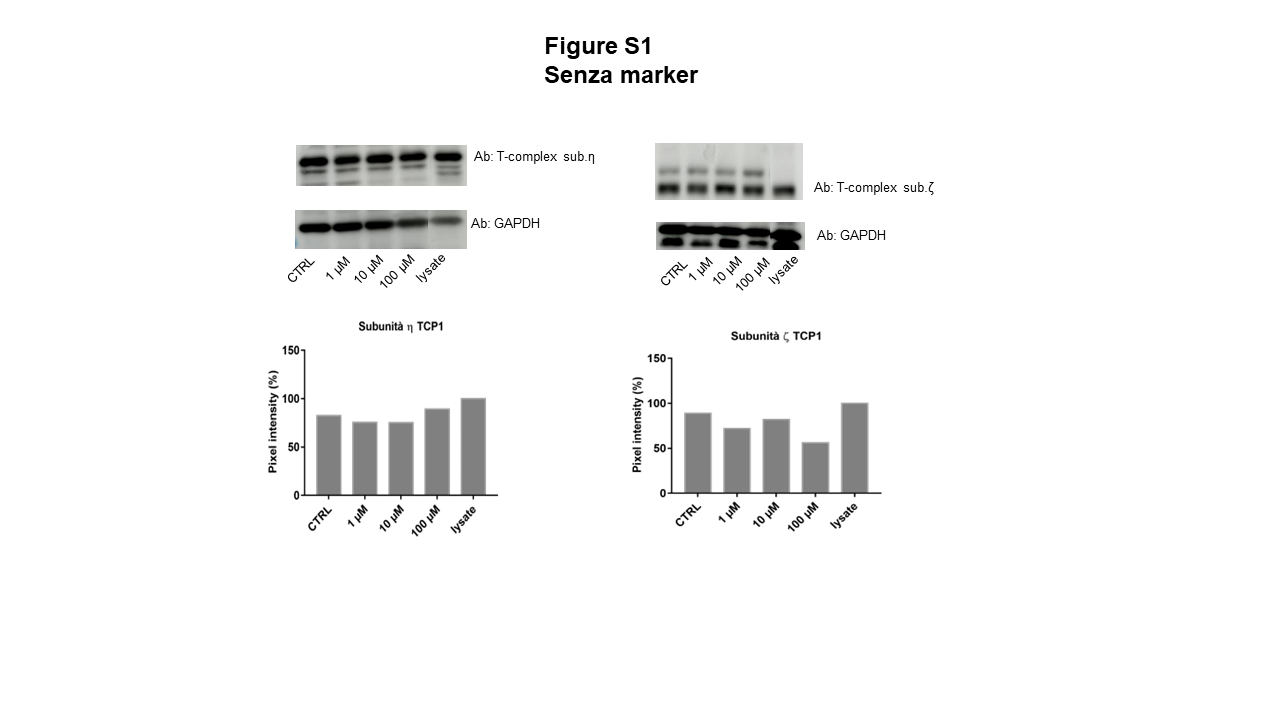
**


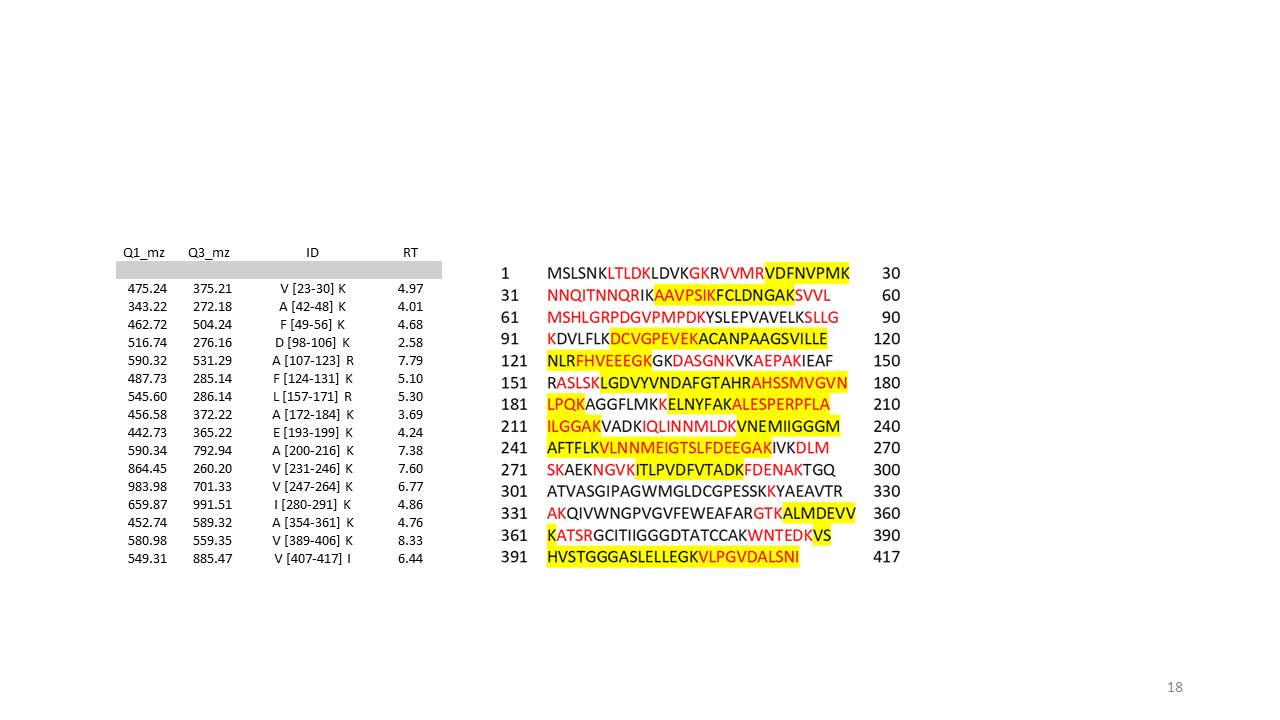
**Figure S1:** lmmunoblotting analysis of one of the DARTS experiments showing that T-complex subunits η and ζ are not protected by TatA. Immunoblottings are reported together with their densitometric analysis. GAPDH is resistant to subtilisin under these experimental conditions and is used as a loading control.

**Figure S2:** MRM quantified PGK1 peptides reported with their Q1 and Q3 m/z value, their length and the retention time in UPLC-MS. In yellow the same peptides reported on PGK1 primary sequence.

|  | **TatA 1/ctrl** | | | **TatA 10/ctrl** | | | **Lysate/ctrl** | |
| --- | --- | --- | --- | --- | --- | --- | --- | --- |
| **ID** | **Fc** | **p-Val** | | **Fc** | **p-Val** | **Fc** | | **p-Val** |
| A-[200-216]-K | 2.02 | 0.60 | 0.56 | | 0.73 | 298.76 | | 0.01 |
| V-[389-406]-K | 1.06 | 0.79 | 1.06 | | 0.76 | 0.76 | | 0.38 |
| L-[157-171]-R | 0.90 | 0.01 | 1.00 | | 0.90 | 4.55 | | 0.01 |
| A-[172-184]-K | 0.97 | 0.82 | 1.06 | | 0.20 | 2.78 | | 0.01 |
| A-[107-123]-R | 0.82 | 0.61 | 0.74 | | 0.65 | 7.25 | | 0.01 |
| V-[247-264]-K | 0.00 | 0.42 | 0.00 | | 0.42 | 9.02 | | 0.01 |
| V-[407-417]-I | 0.22 | 0.31 | 0.66 | | 0.52 | 1.79 | | 0.34 |
| V-[407-417]-I | 0.92 | 0.65 | 0.65 | | 0.27 | 3.55 | | 0.25 |
| I-[280-291]-K | 0.86 | 0.46 | 1.41 | | 0.11 | 3.28 | | 0.01 |
| V-[231-246]-K | 0.91 | 0.83 | 1.00 | | 1.00 | 5.49 | | 0.01 |
| D-[98-106]-K | 0.75 | 0.10 | 1.05 | | 0.87 | 1.05 | | 0.67 |
| F-[49-56]-K | 0.96 | 0.79 | 1.18 | | 0.17 | 3.91 | | 0.01 |
| F-[124-131]-K | 0.90 | 0.63 | 0.93 | | 0.78 | 6.03 | | 0.01 |
| A-[354-361]-K | 1.01 | 0.92 | 0.93 | | 0.53 | 2.30 | | 0.01 |
| A-[42-48]-K | 1.07 | 0.49 | 0.90 | | 0.66 | 1.64 | | 0.03 |
| V-[23-30]-K | 1.05 | 0.40 | 1.05 | | 0.22 | 1.16 | | 0.07 |
| E-[193-199]-K | 1.73 | 0.04 | 2.97 | | 0.01 | 22.79 | | 0.01 |

**Figure S3:** MRM quantified PGK1 tryptic peptides areas after TatA incubation at 1 and 10 µM concentrations. Fc represents the Fold Change as the ratio between the peptide area in presence of TatA over control experiment without TatA. Each Fc is reported with the corresponding p-value. In the last two columns, the ratio of the area of each peptide from the lysate treated only with trypsin over the one treated with subtilisin and trypsin has been reported.


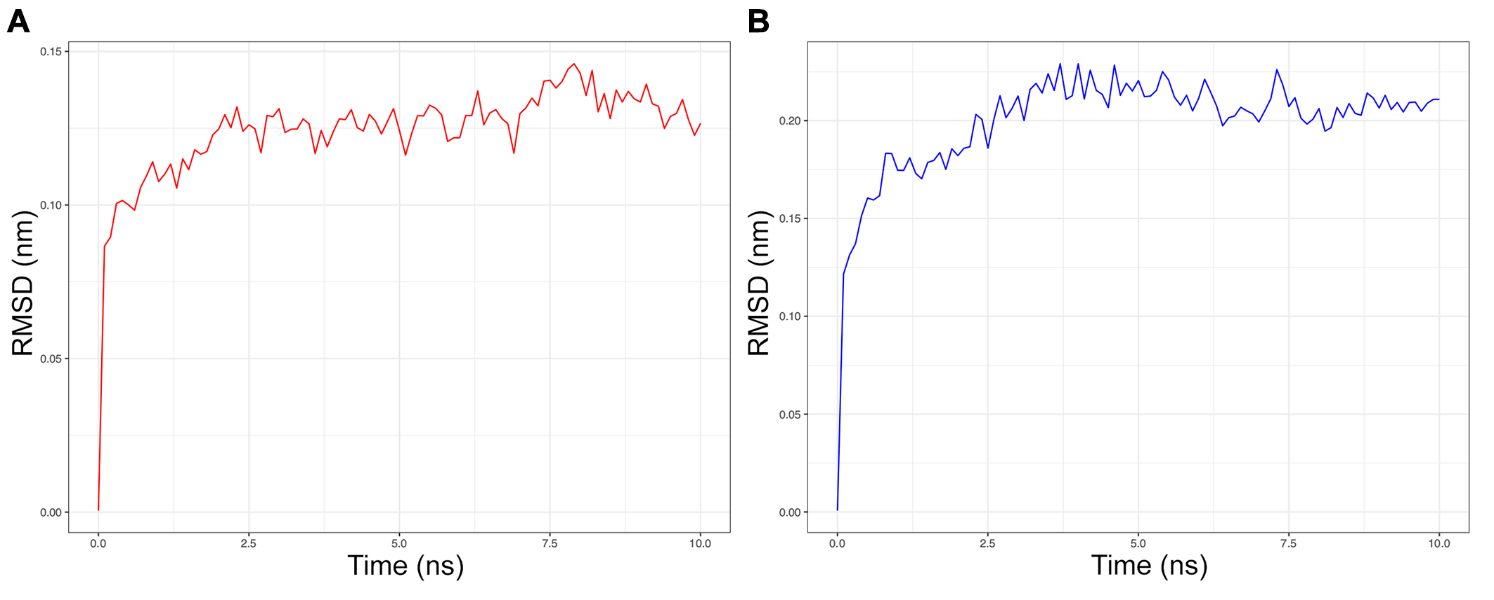
**Figure S4:** Molecular Dynamics analysis of the complex between TatA and PGK1. The RMSD of the complex (A) and of the ligand (B) trajectories with respect to the backbone is shown.


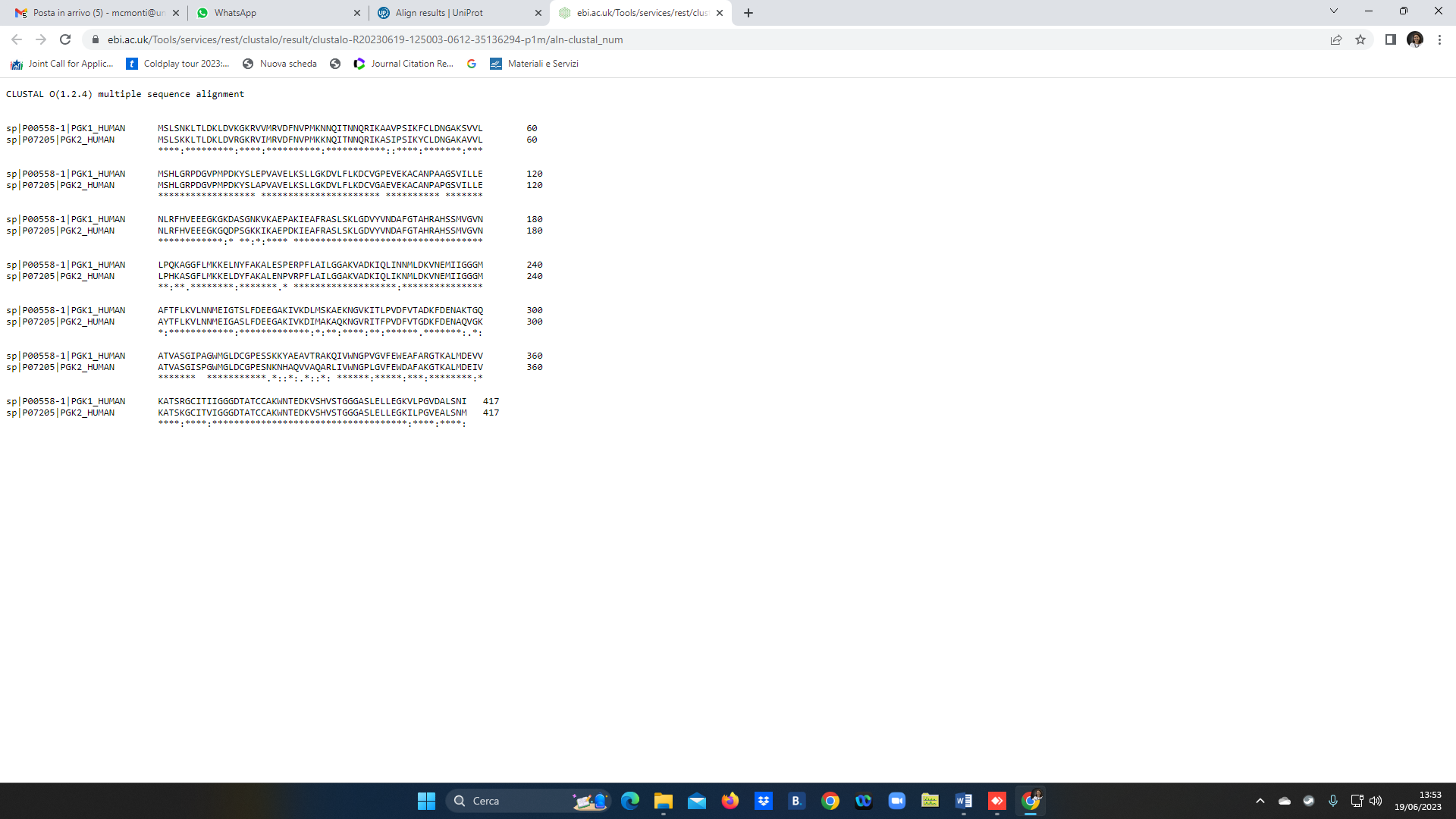


**Figure S5:** Sequence alignment of human PGK1 (Uniprot code P00558) and PGK2 (Uniprot code P07205): the two proteins share 87-88% of sequence identity. The sequence number for P07205 in the alpha fold model is different from the one reported in the sequence alignment: in the alpha fold Ser is the first residue while in the sequence alignment is the second. Accordingly to the Expression Atlas database (<https://www.ebi.ac.uk/gxa/home>), PGK1 is expressed in THP-1 and in KATO III with a good normalized gene expression value, where PGK2 is poorly expressed in both cell lines (under the limit of quantification).


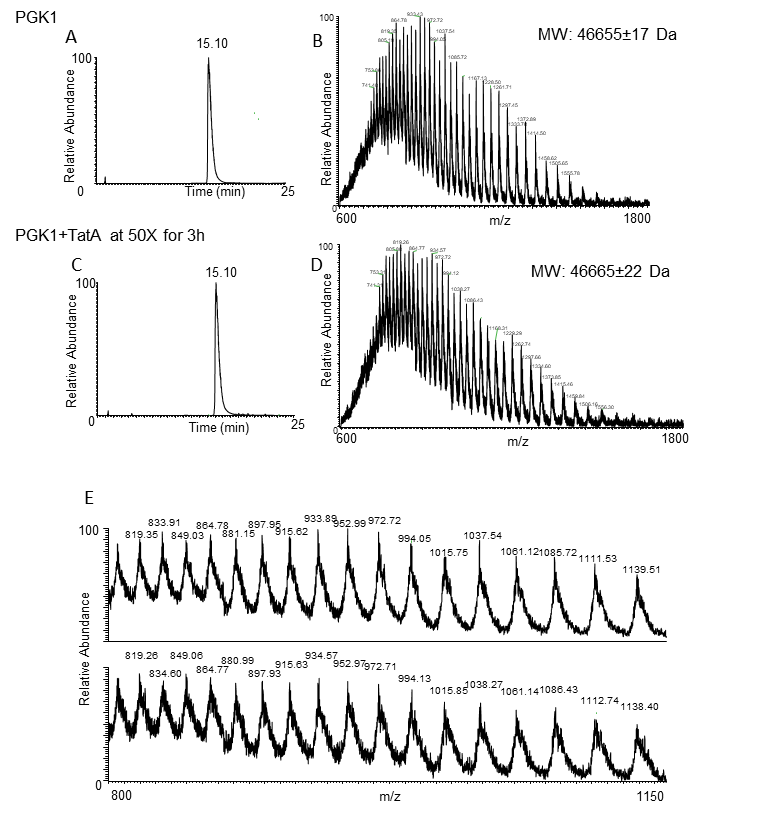


**Figure S6:** In Panel A and B, the LC trace and MS spectrum of PGK1 at 5 µM are reported, together with the deconvoluted MW as obtained using the software ESIProt online. In Panel C and D, the LC trace and MS spectrum of PGK1 at 5 µM incubated for 3 h with TatA at 250 µM at 37°C in PBS buffer are reported, together with the deconvoluted MW as obtained using the software ESIProt online. In panel E, a zoom-in of both spectra are compared: the upper one is relative to PGK1 alone, the lower one to PGK1 incubated with TatA as described.

**
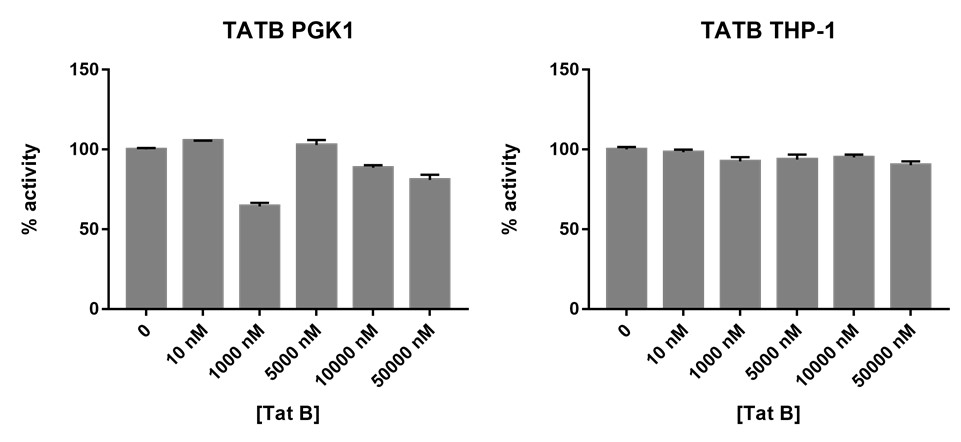
**

**Figure S7:** TatB is unable to inhibit PGK1 activity on the recombinant enzyme and on THP-1 cell lysate. The graphs were build using PRISM software.

**
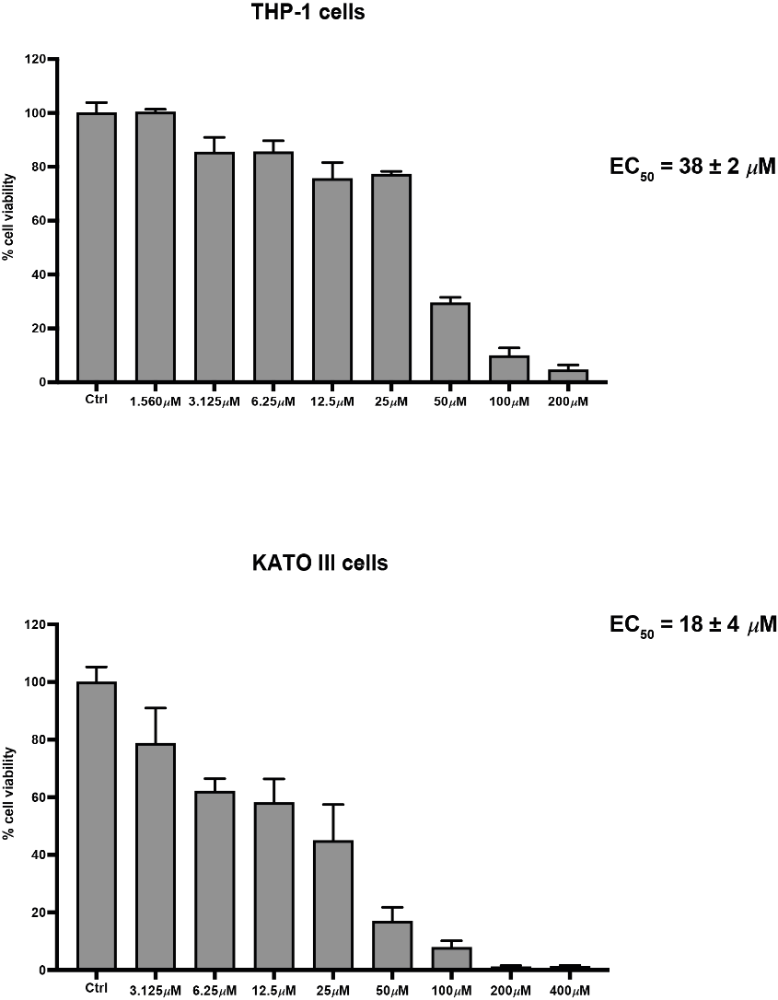
**

**Figure S8: TatA is cytotoxic for THP-1 and KATO III cells.** The EC_50_ value of THP-1 and KATO III cells was evaluated by MTT assay. Cell death and corresponding EC_50_ value after incubation with TatA for 72 h. Each data point represents an average of four independent experiments.
